# In silico identification and validation of vaccine and drug targets in *Coccidioides posadasii* through integrated genomic, proteomic, and molecular modeling approaches

**DOI:** 10.1097/MD.0000000000049169

**Published:** 2026-06-05

**Authors:** Abdullah R. Alanzi, Hattan A. Alharbi, Ramzi A. Mothana, Mohammad K. Parvez, Abdelaaty A. Shahat

**Affiliations:** a Department of Pharmacognosy, College of Pharmacy, King Saud University, Riyadh, Saudi Arabia.

**Keywords:** *Coccidioides posadasii*, Coccidioidomycosis, drug targets, MD simulations, subtractive proteomics, vaccine targets

## Abstract

*Coccidioides posadasii* is a pathogenic fungus endemic to the arid regions of the Americas, causing coccidioidomycosis, a debilitating respiratory infection. *Coccidioides* infection is caused by breathing arthroconidia generated by mycelia that grow in desert soil. Arthroconidia develop inside the lung into massive, multinucleated spherules that are pathognomonic. Despite its significant impact on public health, there remains a paucity of research investigating novel therapeutic approaches to combat this disease. Addressing this research gap, our study employs a multi-faceted approach integrating genomics, proteomics, and computational biology to elucidate the pathogenesis of *C posadasii* and identify potential therapeutic targets. Our methodology involved retrieving genomic data from 5 strains of *C posadasii* and generating core proteomes using OrthoFinder, enabling the identification of extracellular proteins as vaccine candidates and metabolic pathways as drug targets. This approach benefits the research study by providing a comprehensive understanding of the molecular mechanisms underlying *C posadasii* infection, thereby facilitating the development of targeted interventions against valley fever. Our results reveal promising drug-like candidates for drug targets and vaccine targets CID:123631, CID:193962, CID:3045233, CID:6741, CID:214348, CID:11450633 with the identified proteins demonstrating potential efficacy against *C posadasii*. As we embark on the journey towards translating these discoveries into clinical practice, our findings offer hope for a future where effective treatments for coccidioidomycosis are within reach, promising relief to countless individuals afflicted by this devastating disease.

## 1. Introduction

In the arid regions of North, Central, and South America, a pathogenic fungus known as *Coccidioides posadasii* produces endemic and epidemic cases of coccidioidomycosis.^[1]^ Notably, there are distinct species distributions within the genus *Coccidioides*: *Coccidioides immitis*, which is found exclusively in California, and *C posadasii*, formerly known as non-California *C immitis*, which is found primarily in Texas, Arizona, and the areas of endemicity in Central and South America. The disease has been emerging in areas to which it is not endemic and increasing in incidence among immunocompromised hosts.^[2]^ The 2 species are physically identical and have approximately 90% homology in their predicted proteins.^[3]^ Infection with *Coccidioides* species is caused by inhaling arthroconidia produced within mycelia that grow in desert soil. Arthroconidia differentiate inside the lung into enormous, multinucleate spherules, which are pathognomonic. When mature spherules burst, endospores emerge, develop, and differentiate into the next generation of spherules. Infection causes a wide variety of symptoms, from none (asymptomatic infection diagnosed by skin test positivity) to mortality via extrapulmonary dissemination, depending on the host’s immune condition and unknown genetic factors.^[4]^

*C posadasii* is a fungal respiratory infection of humans that can cause illness in immunocompetent people. Coccidioidomycosis infections can range from mild to severe. It is commonly described as either a chronic condition that takes months to resolve or an asymptomatic infection that can return several years after the initial shock.^[5]^ Valley fever, also referred to as coccidioidomycosis, was initially identified in Argentina in the late 1800s by Dr Alejandro Posadas.^[6]^ After it was discovered that the causative agent was a dimorphic fungus, most of the sickness being asymptomatic or mild, the causal agent was originally believed to be a protozoan that produced severe disease.^[7]^ Coccidioidomycosis is caused by 2 species, *C immitis* and *C posadasii*.

Serologic tests cannot discriminate between the 2 species, although genetic polymorphisms can, and changes in growth parameters have been found.^[8,9]^ *C posadasii* has a greater population and is more diversified than *C immitis*. The main distinction between the 2 species is their geographical spread. *C immitis* is primarily found in the deserts of Central and Southern California (including Baja California), whereas *C posadasii* is primarily found in the deserts of Nevada, Arizona, New Mexico, West Texas, Mexico, and Central and South America, implying that significant geographic barriers existed when the species diverged from a common ancestor.^[10]^ Geographic overlap between the 2 species is also found in Southern California and Baja California.^[11]^ Genetic study of various genetic markers revealed 2 monophyletic clades.^[8]^ Subsequent population genetic/genomic investigations revealed that *C immitis* has at least 2 populations in the western United States, while *C posadasii* has 3 populations spread across the American continents.^[10,12,13]^ Given the significant frequency of autapomorphic mutations between *Coccidioides* species and isolates within species, phenotypic diversity is expected.^[14]^ However, little research into phenotypic differences has been conducted. A recent investigation found that *C immitis* in vitro spherules developed synchronously, whereas *C posadasii* isolates did not.^[15]^ Inhaled arthroconidia, the asexual propagule that forms in the environment, gives rise to the first spherule. During this stage of life in the environment, the fungus starts off as unremarkable mycelia and develops into alternating arthroconidia as it ages. Although the precise environmental conditions needed for growth and maturation are uncertain, evidence points to the involvement of precipitation and keratin sources.^[16,17]^ After infection is established, the host mostly experiences the spherule life stage, during which mature endospores are released by rupture of the outer cell wall and internal endospore development. Endospores can differentiate into new spherules on their own, and host immune cells are able to identify and absorb them.^[18,19]^ The spherule might rupture the host cell once it has grown and matured to the point where it cannot be absorbed. *Coccidioides* is a disease that can live both inside and outside of cells. Although the sexual stage of the life cycle is unknown, it seems to occur frequently.^[20,21]^

As we investigate the multifaceted determinants of Coccidioidomycosis outbreaks, the current increase in case numbers is due to a combination of host and environmental variables. Modifiable hazards associated with recreational/occupational activities and travel to highly endemic locations are examples of host factors, as are non-modifiable risks such as aging and complex underlying health disorders, which are summarized below. Natural events and climate change are examples of environmental factors. Lastly, the increasing number of reported cases may be a result of improved case detection. The risk of infection is increased by human activities that permit the fungus to spread via the air.^[22]^ As evidenced by the outbreak at Dinosaur National Park,^[23,24]^ as well as on solar power farms,^[25,26]^ archaeology, construction, and firefighting especially when using hand tools and working in dusty conditions have all been connected to infection.^[27]^ Smith initial studies in the 1940s^[28]^ demonstrated that military personnel stationed in endemic areas are also more vulnerable, and more recent research has confirmed the continued risk for military personnel engaged in outdoor military training.^[29,30]^ Environmental factors are also significant contributors to increased coccidioidal rates. Incident infections have been exacerbated by the development of traditionally sparsely populated areas into large metropolitan centers, including Phoenix, Arizona. The enormous influx of people into these areas, along with the nearby recreational activities on open soil, may have tipped the scales in favor of increased infections, even though urbanization produced paved streets and buildings that covered previously open desert soil (with less potential exposure to airborne arthroconidia. Additionally, many jails have been built in areas where the disease is prevalent (such as California’s central valleys), where there have been significant outbreaks among the inmates’ populations where minority ethnicities are overrepresented and who are more likely to experience symptoms and severe illness.^[25]^ Other variables are natural events and the climate. After heavy winter or spring rains, infection rates increase throughout the arid summer months. The regional distribution and case numbers may therefore change because of climate change, especially dry summers. It has been suggested that endemic regions may move north due to global warming, increasing the fungus’s geographic spread.^[31]^

To find some medical solutions and some potential drug and vaccine candidates to cure this disease, we have designed this efficient experimental design to conduct research. This study represents a comprehensive investigation into the genomic and proteomic landscape of *C posadasii*, shedding light on its virulence factors, potential drug targets, and vaccine candidates. By employing a multi-faceted approach, we meticulously curated and analyzed genomic data retrieved from National Center for Biotechnology Information (NCBI), leveraging advanced bioinformatics tools and methodologies. Through the rigorous application of OrthoFinder,^[32]^ we identified core proteomes and nonhomologous proteins across multiple strains, laying the foundation for understanding strain-specific variations and evolutionary dynamics. Our findings underscore the importance of considering host–pathogen interactions in the context of coccidioidomycosis pathogenesis, with implications for the development of targeted therapeutics and preventive strategies. Moreover, our study offers novel insights into the subcellular localization and essentiality of key proteins, providing valuable clues for understanding the pathophysiology of *C posadasii* infection. By elucidating the spatial distribution of virulence factors and essential genes, we aim to unravel the intricate mechanisms underlying fungal pathogenicity and host immune evasion. Furthermore, our identification of potential vaccine targets and drug candidates presents promising avenues for the development of efficacious interventions against coccidioidomycosis. The integration of structural prediction and molecular docking analyses enhances our understanding of protein–ligand interactions, facilitating rational drug design and optimization. Moreover, this study stands as a testament to the power of interdisciplinary collaboration, bridging the gap between bioinformatics, microbiology, and clinical research to tackle pressing public health challenges. As we embark on the next phase of our investigation, armed with a deeper understanding of *C posadasii* biology and pathogenesis, we are poised to catalyze the development of innovative diagnostic tools, targeted therapies, and preventive measures against valley fever. Indeed, the implications of our work extend far beyond the confines of academia, offering tangible solutions to alleviate the burden of coccidioidomycosis on affected individuals and communities worldwide. In this era of precision medicine and data-driven healthcare, our bioinformatic endeavors hold the promise of transforming the landscape of infectious disease management, ushering in a new era of personalized interventions and improved outcomes for patients battling valley fever.

## 2. Materials and methods

### 2.1. Data retrieval

NCBI was used to retrieve 5 strains of *C posadasii* which are as follows, GCA_000151335.1, GCA_018416015.2, GCA_020976795.1, GCA_020976775.1, and GCA_000150055.1. Proteomes of 2 out of 5 strains were not available on NCBI. These strains are as follows: GCA_020976795.1 (Strain: 3796), GCA_020976775.1 (Strain: 2566). To get proteomes of these 2 genomes, Augustus v3.4.5 tool was used. The model organism which was used in Augustus was *C immitis* because the morphology of *C posadasii* and *C immitis* is identical, and more than 90% homology is expected between their predicted proteins.^[3]^ In Augustus, a soft-masking feature was used. Genes were predicted on both strands and a complete gene model was used. No extrinsic information was used and neither prediction sequence range was specified.

### 2.2. Generation of core proteome and identification of nonhomologous proteins

A popular orthology analysis tool, OrthoFinder^[32]^ uses a graph-based algorithm to identify orthologous groups in various species. It creates a similarity matrix and uses the Markov Cluster algorithm to identify orthologous groups, offering a reliable way to determine orthology.^[32]^ The parameters which were used in OrthoFinder are: inflation value was 1.50 5 selected based on benchmarking studies by Emms and Kelly^[32]^ showing optimal performance for proteomes ande-value was 1e−5. The orthologous protein identification and core proteome generation of 5 distinct strains of *C posadasii* were accomplished by utilizing the OrthoFinder tool. The drug and/or vaccine candidate must not be homologous to human proteins to prevent autoimmunity. After the core proteome was acquired, it was compared against the host proteome database using the BlastP algorithm. BlastP is a program that finds homologous proteins by matching protein sequences to a protein database and analyzing sequence similarity.^[33]^ An e-value of 1e−5 with ≤ 25% sequence identity cutoff was used to filter low complexity regions and human homologs for getting meaningful results. Therefore, to prevent autoimmunity reactions or excessive drug toxicity to host cells, only proteins with the least amount of host identity were obtained.

### 2.3. Identification of subcellular location and essentiality

The proteins identified as conserved and nonhomologous to the host (human) from OrthoFinder and BlastP analyses were subjected to subcellular localization prediction using the Cello tool.^[34]^ A computational tool Cello can be used to predict the subcellular location of proteins and provide details on their cellular compartmentation. Support vector machines trained by numerous feature vectors based on n-peptide compositions are used in this method. After obtaining results, cytoplasmic proteins and extracellular proteins were selected for further analyses. The selection was based on statistical parameters such as compartment, score, and rank, ensuring the inclusion of top and statistically significant proteins. Once subcellular locations of proteins were identified with Cello v2.5, the essential genes of the *C posadasii* strains were predicted using the web server Geptop 2.0^[35]^ and only the most significant genes were selected based on class and essentiality score provided by Geptop 2.0. By combining orthology and phylogeny and comparing query protein sets with experimentally determined essential gene datasets from the Database of Essential Genes database,^[36]^ Geptop 2.0 offers a platform for essential gene discovery across bacterial species.

### 2.4. Identification of vaccine and drug targets

Best hit proteins chosen for vaccine targets which are extracellular proteins were analyzed using TMHMM^[37]^ and Uniprot^[38]^ for protein features and information. The 35 proteins were chosen based on statistics and results from TMHMM and Vaxijen 2.0^[39]^ web server with a threshold of 0.6. A threshold of 0.6 indicates the antigenic score, which reflects the likelihood that a protein is an antigen capable of eliciting an immune response. A higher threshold increases the stringency of antigen prediction, reducing the chances of false positives but potentially missing some true antigens. For the identification of drug targets, a small genomic subtraction technique was used to identify the unique metabolic pathways. The metabolic pathways of the pathogen and the listed host *Homo sapiens*, were examined to uncover exclusive processes found in the pathogen. Next, using BLASTp in the KEGG Automation Annotation Server (KAAS) against the KEGG database,^[40]^ the query proteins were functionally annotated. Using the bi-directional best hit approach in KAAS, the identified metabolic proteins’ KEGG orthologs were allocated. In a similar manner, specific proteins from unique pathways were assessed using BLASTp with an E-value of 10−e5 against the DrugBank database^[41]^ to ascertain their potential as therapeutic targets, leading to the identification of new targets. The Food and Drug Administration-approved custom drug target dataset was used to examine the proteins. Proteins exhibiting an 80% or higher similarity frequency with the Food and Drug Administration-approved DrugBank database were deemed suitable candidates for drug development.

### 2.5. 3D structure prediction and validation

3D structures of the proteins were predicted which were not already available on Uniprot or Protein Data Bank generated by using Swiss-Model tool also known as structure modeling server.^[41,42]^ A0A0J8S610.1.A, an already predicted model from *C immitis* H5384 was used as a template for the structure prediction for A0A0J8S610 in results section in Table S3, Supplemental Digital Content. J3KLF1.1. Glutathione S-transferase, an already predicted model from *C immitis* strain RS was used as a template for the structure prediction for E9CRD4. A0A0J8RAY9.1. A Glutathione S-transferase, an already predicted model from *C immitis* strain 3703 was used as a template for the structure prediction for A0A0J8RAY9. The Galaxy Refine online server was used to further refine and modify the 3D predicted structures. The Galaxy Refine server reshapes the side chain before performing structural reassembling and overall structural assessment using molecular dynamics (MDs).^[43]^

### 2.6. Molecular docking analysis and absorption, distribution, metabolism, excretion and toxicity

Before performing molecular docking simulation, the chosen proteins were prepared to make it a perfect receptor for molecular docking. Structures of receptors and ligands were prepared with PyRx tool^[44]^ and minimization was performed. Polar hydrogen molecules and partial charged were added to the 3D structured protein by Discovery Studio tool.^[45]^ Active site of the receptor was predicted by DoGSiteScorer^[46]^ online server. This site was selected as site specific docking in PyRx so that the program should only bind ligands in that specific active site during the process. A database of 3 thousand natural drug-like compounds was used against the predicted binding site of receptor to dock downloaded from ZINC database. Based on root mean square deviation (RMSD) and docking score, the best interacting ligand molecules with the targets were filtered out. Right after docking studies, a thorough absorption, distribution, metabolism, excretion and toxicity using SwissADME^[47]^ was conducted at top candidates for screening of highly drug-like molecules. So, top 1 compound was selected for each target candidate from vaccine and drug targets. Furthermore, the shortlisted compounds from RMSD and binding affinity were evaluated based on Lipinski rule of 5. PyMOL v 2.5^[48]^ and Discovery Studio v 2021 client was used for 2D interactions visualizations.^[45]^

### 2.7. MD simulations

To assess the stability and validate the docking results, MD simulations were performed using Desmond to replicate physiological conditions for the protein–ligand complexes over 100 nanoseconds.^[49]^ The selected compound-receptor complexes underwent comprehensive stability testing through MD simulations. The protein–ligand systems were prepared through a sequential workflow involving preprocessing, structural optimization, and energy minimization steps prior to MD simulation initiation. Energy minimization was performed using the OPLS_2005 force field parameters.^[50]^ Each system was solvated within an orthorhombic simulation box (10 Å × 10 Å × 10 Å) filled with TIP3P water molecules.^[51]^ Physiological conditions were established by neutralizing the systems with appropriate counter-ions and maintaining a 0.15 M NaCl concentration. The simulations employed an NPT ensemble^[52]^maintained at 300 K temperature and 1 atm pressure. Following system equilibration through a relaxation protocol, production runs were executed for 100 ns using a 2 fs timestep. Trajectory data were captured at 40 ps intervals throughout the simulation period to enable comprehensive post-simulation analysis of system behavior and binding stability.

## 3. Results

### 3.1. Phase I: subtraction

The proteomes of 5 strains of *C posadasii* which were retrieved from GenBank database^[53]^ hosted by NCBI produced 6578 proteins overall. The information of genomes retrieved from GenBank NCBI is given in Table S1, Supplemental Digital Content. A total of the 3074 proteins out of 6578 proteins were deemed as host nonhomologous proteins. Cello tool utilizes a machine learning-based approach, combining various sequence-derived features, to predict the subcellular localization of proteins (Table S2, Supplemental Digital Content). It considers information such as amino acid composition, physicochemical properties, and functional motifs to make predictions.^[54]^ And predicted genes were subjected for essentiality analysis using Geptop 2.0 webserver with a threshold of 0.00001. Geptop 2.0 identified 343 essential proteins from 369 cytoplasmic proteins based on lower to higher score of essentiality and 26 proteins with zero essentiality. For secreted proteins, Geptop 2.0 identified 142 essential proteins out of 150 proteins based on lower to higher score of essentiality and 8 proteins with zero essentiality (Table 1; File S1, Supplemental Digital Content).

**Table 1:** The reduction in the number of proteins in each subtractive approach.

Sr. no.	Step	Number of proteins reduced
1	Core Proteome Generation	6578 (File S1, Supplemental Digital Content)
2	Human Nonhomologous Proteins	3074
3	Cytoplasmic Proteins/Secreted Proteins	369/150
4	Essential Cytoplasmic/Secreted Proteins	343/142
5	Cytoplasmic Proteins with Unique Metabolic Pathways/Top secreted proteins from TMHMM 2.0	21/35
6	Cytoplasmic Druggable Proteins/Secreted proteins from Vaxijen as probable ANTIGEN	3/17 (File S1, Supplemental Digital Content)
	Total drug and vaccine candidates	6

### 3.2. Phase II: drug and vaccine candidate prioritization

Thirty-five top extracellular proteins chosen for vaccine targets proteins were analyzed using TMHMM and Uniprot for proteins features and information. The 3 proteins were chosen based on statistics and results from TMHMM and Vaxijen 2.0 webserver with a threshold of 0.6. E9D8Y3 encoded by E9D8Y3_COCPS having 100% similarity, having a key role as “Lyase Activity.” E9DEI6 encoded E9DEI6_COCPS with 100% with having essential family domains of Calcium dependent cell adhesion molecule-1 1 hit, CAD_C_sf, and membrane bd. A0A0J8S610 encoded by Cyanovirin-N domain-containing protein having similarity 100%. It has an importantdomain which is a signal domain, a peptide usually presents at N-terminus of proteins, and which is destined to be either secreted or part of membrane components. Similarly for the identification of drug candidates, the remaining 343 proteins were subjected to KEGG and KAAS to predict the unique pathways. These proteins engaged in 106 pathways from which only 21 proteins engaged in unique pathways. DrugBank predicted only 3 top proteins as druggable candidates (Table S3, Supplemental Digital Content). E9CRD4 encoded by Glutathione transferase having 98.4% similarity, having a key role as “Transferase Activity.” A0A0J8RAY9 encoded by Glutathione S-transferase having 96.7% similarity, having a vital role as “Transferase Activity.” E9CWQ6 encoded by Catechol dioxygenase having 100% similarity, having a key role as “catechol 1,2-dioxygenase activity,” “ferric iron binding,” and “catechol-containing compound metabolic process.”

### 3.3. Molecular docking analysis

**Figure 1.**
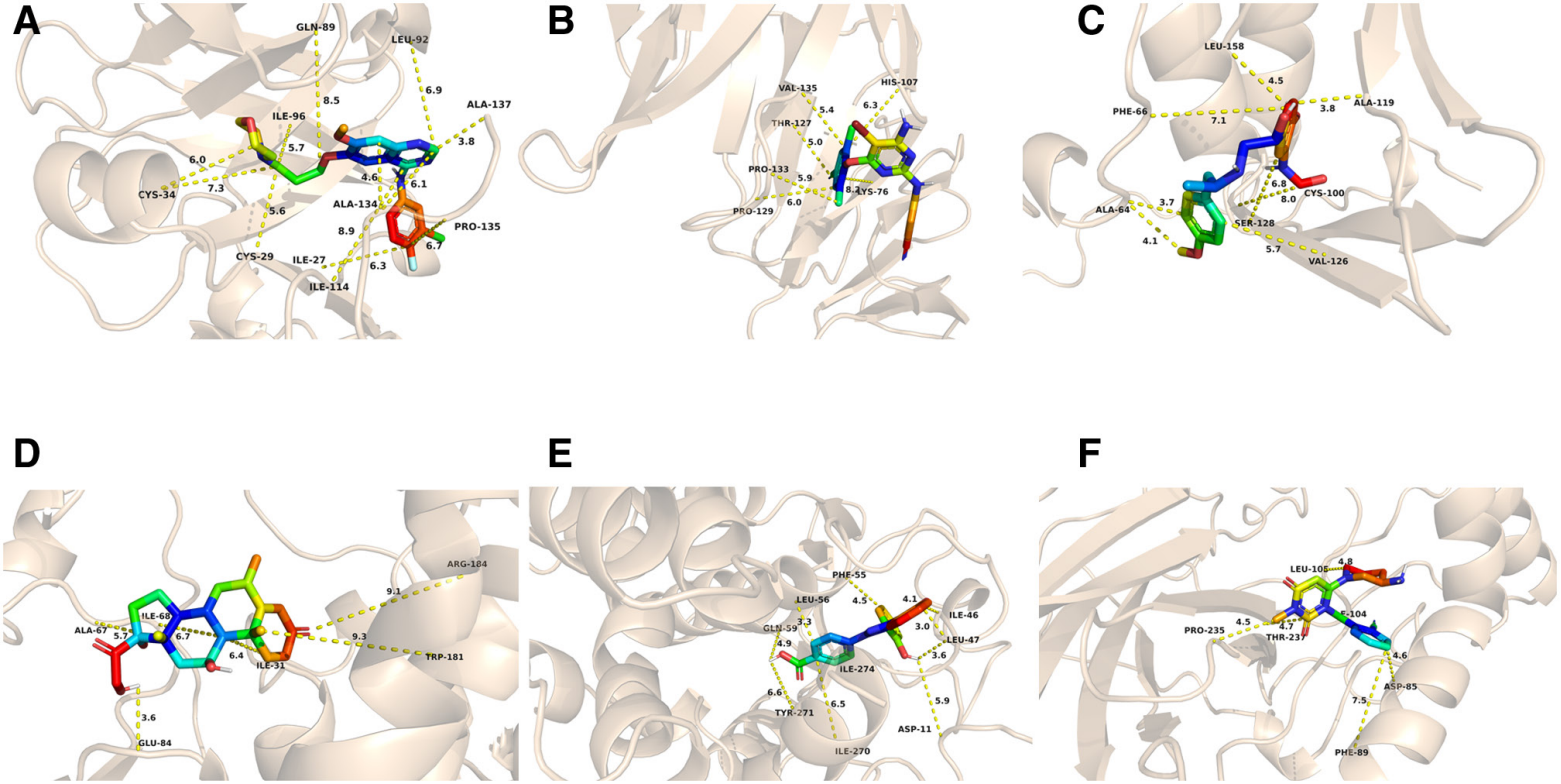
The chosen proteins for vaccine and drug targets along with their docked complexes, the above figure shows the exposed ligand binding site with an attached ligand. (A) The protein is E9D8Y3 and attached ligand is CID:123631 (B) The protein is E9DEI6 and attached ligand is CID:193962. (C) The protein is A0A0J8S610 and attached ligand is CID:3045233. (D) The protein is E9CRD4 and attached ligand is CID:6741. (E) The protein is A0A0J8RAY9 and attached ligand is CID:214348. (F) The protein is E9CWQ6 and attached ligand is CID:11450633.

**Figure 2.**
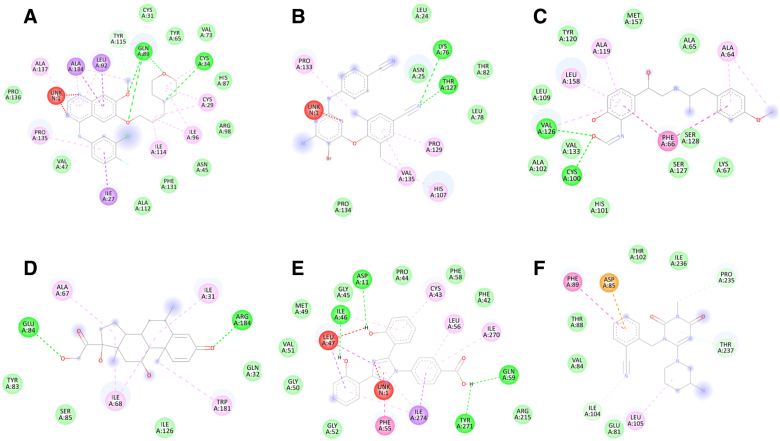
The figure represents the 2D interaction of ligand with the receptor, its clashes and exposure to the receptor’s surface and amino acids residues. The receptor E9D8Y3 and its ligand CID:123631 is being representing by figure (A). The receptor E9DEI6 and its ligand CID:193962 is being representing by figure (B). The receptor A0A0J8S610 and its ligand CID:3045233 is being representing by figure (C). The receptor E9CRD4 and its ligand CID:6741 is being representing by figure (D). The receptor A0A0J8RAY9 and its ligand CID:214348 is being represented by figure (E). The receptor E9CWQ6 and its ligand CID:11450633 is being represented by figure (F). The red color residues represent unfavorable bump, hot pink represents Pi-Pi stacked, light pink represents Pi-Sigma and Pi-Alkyl, hot green represents Conventional hydrogen bond and light green represents van-der Waals, and orange represents Pi–Anion interactions.

After the preparations of protein as a receptor to subject it to molecular docking process, first the active site for ligand binding was predicted by DoGSiteScorer and then it was validated by DoGSiteScorer. After the docking process, strict criteria were implemented to choose the best hits to ensure the selection of best drug-like compounds out of results. Statistics such as binding affinity (kcal/mol), RMSD, and Lipinski rule of 5 were considered strictly. The lower the binding affinity and RMSD, the more it indicates a stronger Receptor-Ligand complex. The scores and statistics for CID:123631, CID:193962, CID:3045233, CID:6741, and CID:214348, CID:11450633 ligands are provided in Table S4, Supplemental Digital Content. CID:123631 is showing great interactions such as formation of hydrogen bond with Leu92, basic interaction of Ile96 to ligand’s oxygen atom and other interactions of ligand with receptor’s residues are with Ile114 and Cys34 with a greater ligand exposure (Fig. 1A). In ligand CID:193962, Thr127 is directly interacting as sidechain acceptor interaction with ligand, Val135, His107, and Pro133 has greater exposures with ligand’s atoms. Lys76 is forming a hydrogen bond with ligand’s atoms (Fig. 1B). CID:3045233 ligand is showing greater interactions with its receptor such as Ala119 forming an arene-H bond with ligand. Phe66, Leu158, Ala64, Cys100, and Val126 are interacting with ligand atoms as backbone acceptors and donors (Fig. 1C). CID:6741 is proving itself to be a great drug-like compound for receptor. Its atoms are interacting as sidechain acceptors with Arg184, Trp181, Ile31, and Ala67 (Fig. 1D). In receptor’s complex with CID:214348, Cys43 being the nearest residue is interacting as sidechain acceptor with the ligand’s OH molecule. Leu47, Phe55, Leu46, Leu47, Leu56, and Gln59 are having exposures and interactions with ligand’s atoms indicating the best binding with receptor (Fig. 1E). CID:11450633 is also a very promising drug-like candidate and ligand, Thr237 is directly interacting as a backbone acceptor with ligand’s NH_2_ molecule, Leu105 is interacting with ligand’s Oxygen atom as sidechain acceptor (Fig. 1F). Pro235, Thr237, Asp85, Phe89, Leu105 are interacting as donors and acceptors. The hydrogen bonds distances, while the molecular interactions of compounds are shown in Figure 2A–E.

### 3.4. MD simulations

#### 3.4.1. Root mean square deviation

**Figure 3.**
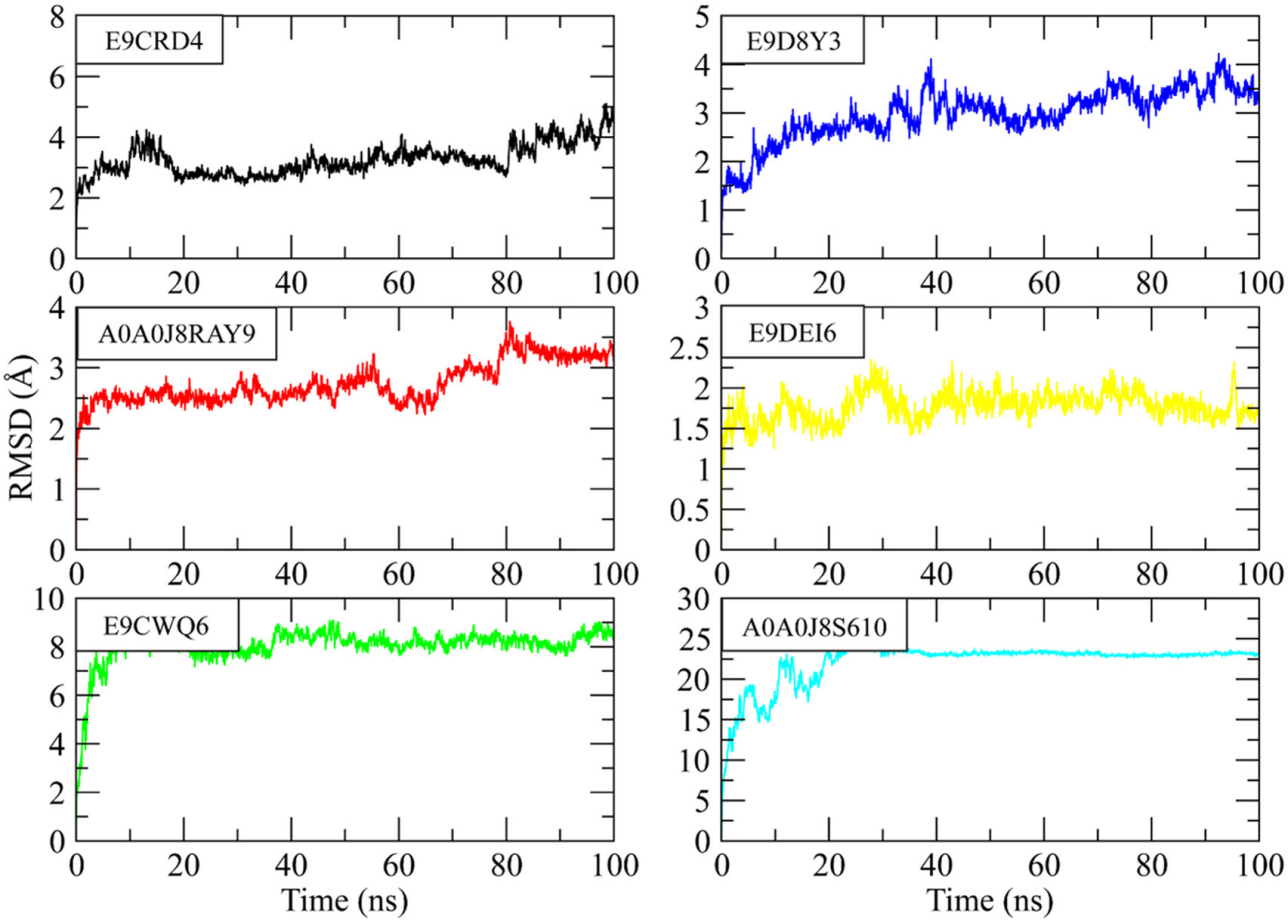
The computed RMSD profiles of the complexes during a 100 ns simulation. RMSD = root mean square deviation.

The binding stability of compounds against the receptor was investigated using MD simulation, which lasted for 100 ns. The RMSD of the carbon alpha (C) atoms was computed to analyze the various deviations and overall structural changes that occurred during the simulation.^[55]^ The RMSD for complex *E9CRD4* started at around 2 Å and increased to 4 Å at 10 ns. It then ranged between 2 and 4 Å throughout the rest of the simulation. The RMSD profile for this compound was highly stable from 20 to 80 ns, deeming it a very significant candidate. Complex *E9D8Y3* started at around 1 Å and increased progressively to about 4 Å at about 40 ns. It was usually due to ligand’s binding conformational changes. Because there were no sudden sharp and higher peaks, it seems ligand was very stable inside the protein’s active site. After then, it decreased to 3 Å, where it remained until the end of simulation. Complex *A0A0J8RAY9* started at 2 Å and increased to 4 Å at 80 ns. It stayed in this range all the way through the simulation. Complex *E9DEI6* had a stable RMSD; it begins at around 1.5 Å and fluctuates little throughout the simulation. There were no major fluctuations seen for *E9DEI6* throughout the 100 ns MD simulations. Complex *E9CWQ6* began at around 2Å, increased gradually to a peak at about 8 Å, and persisted until the simulation was completed. Lastly, for complex *A0A0J8S610* started at 5Å, increased to 25Å, and stayed in this range all the way until the simulation’s end. The start from 5 Å rising up to 25 Å suggests that the complex was equilibrating during this duration, but once it was out of equilibration the RMSD profile was very stable until the end. These results provide insight into the structural changes that have occurred during the simulation; typically, a complex with a lower RMSD value is more stable (Fig. 3).

#### 3.4.2. Root-mean square fluctuation (RMSF)

**Figure 4.**
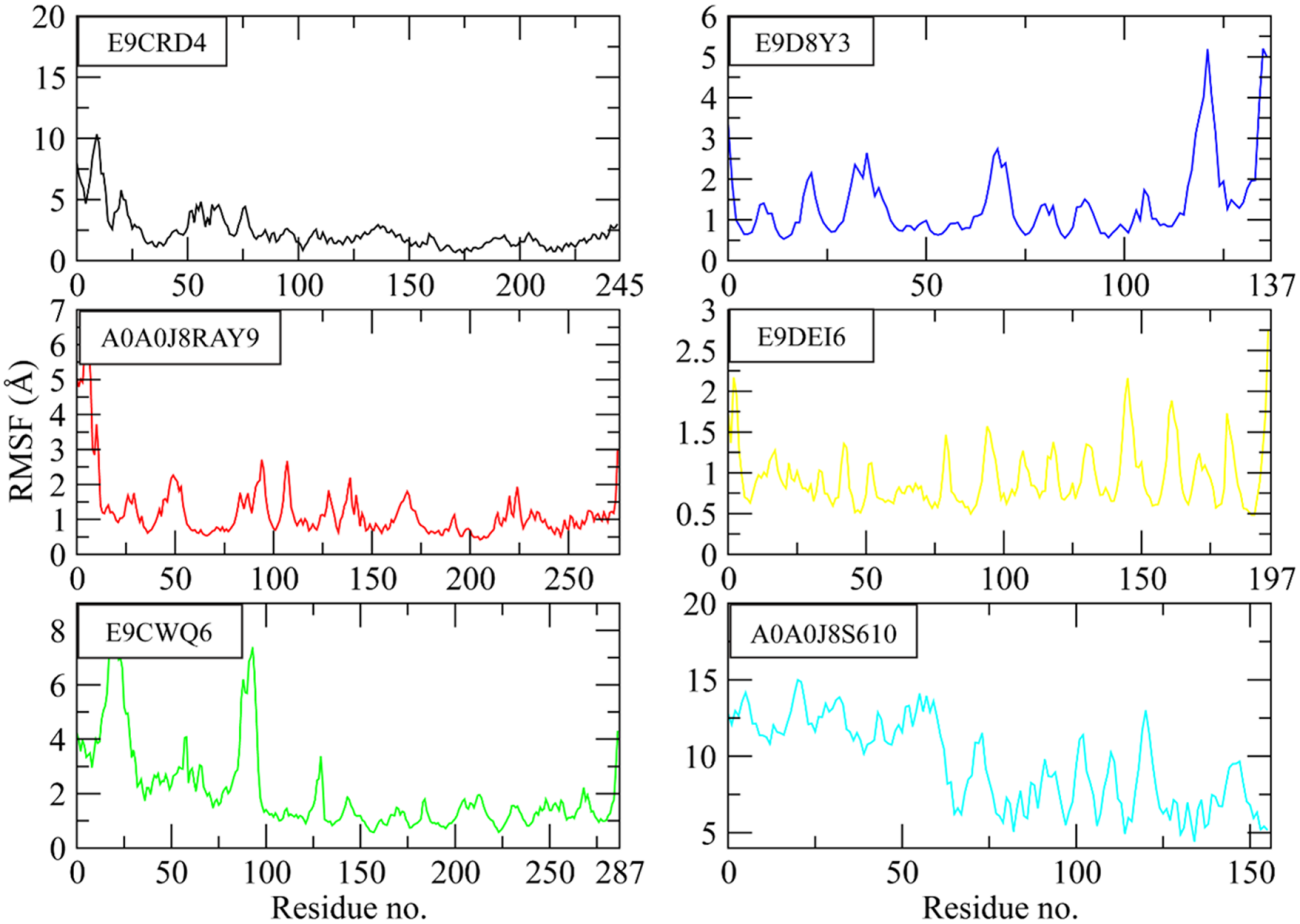
The RMSF of the residues for all the complexes from 100 ns MD simulations. MD = molecular dynamic, RMSF = root mean square fluctuation.

RMSFs approaches have been used to quantify fluctuations in proteins while they are linked to ligands. The RMSF values provide a detailed description of the mobility and flexibility of each protein residue across the simulated period. The RMSF values for complex *E9CRD4* vary across different residue counts, demonstrating the flexibility and mobility of the protein. Most of the residues varied, although not by more than 5 Å, with the initial residues exhibiting the most variation, which are approximately 10 Å. Complex *E9D8Y3* residue RMSF values varied, although not by more than 3.0 Å. The loop parts, however, have higher RMSF values, around 5 Å. Complex *A0A0J8RAY9* RMSF value varied by no more than 3 Å. The initial residues, however, have greater RMSF values that are around 6 Å. The RMSF values for compound *E9DEI6* are stable, fluctuating slightly between 1 and 2 Å throughout the residue numbers. Complex *E9CWQ6* showed varying RMSF values, although not by more than 4.0 Å. On the other hand, the loop sections exhibit larger RMSF values, around 8 Å. Finally, complex *A0A0J8S610* had greater RMSF values, ranging from 5 to 15 Å. These findings give information on each protein residue’s mobility and flexibility over the simulated time. Greater RMSF values for the loop segments signal that there may be more dynamic and variable interactions with the ligands. While most protein residues exhibited very minor alterations, loop regions showed noticeably more flexibility. Overall, the RMSF findings give support to the idea that proteins and ligands interact to create stable complexes (Fig. 4).

#### 3.4.3. Protein–ligand contacts

**Figure 5.**
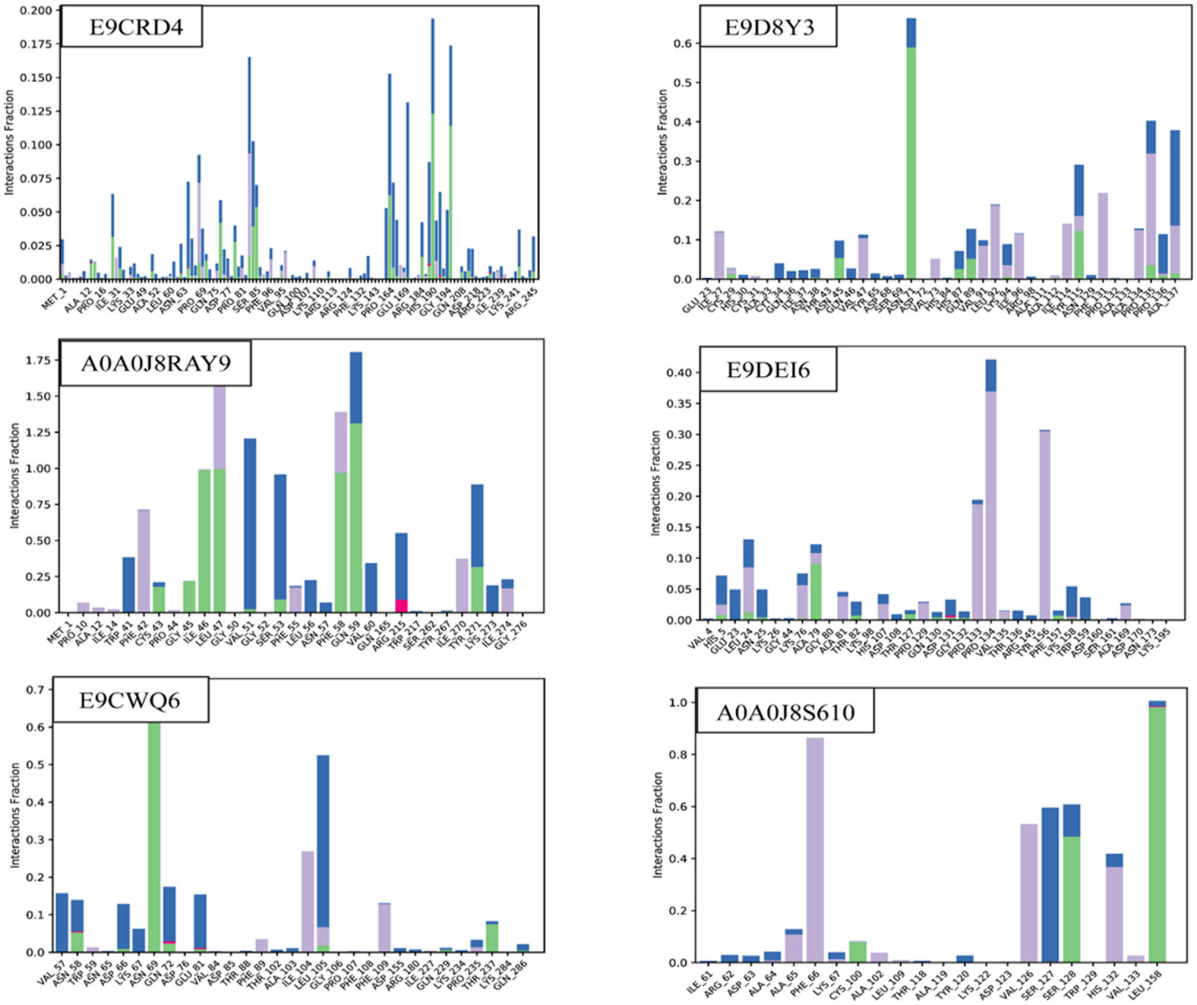
The protein ligand interaction fractions between a protein and a ligand during molecular dynamics simulation.

The MD simulation analysis revealed that the most significant forms of interactions between the ligands and the protein were hydrophobic, hydrogen, and ionic bond interactions. These interactions maintain and modify the functional properties of the protein–ligand complex. The compound *E9CRD4* has been observed to form significant hydrogen bonding interactions with residues such as Ala12, Pro16, Ile31, Gln75, Pro81, Ser85, Pro164, His190, Gly194, Lys241, and Arg245.The residues in the *E9D8Y3* compound that showed hydrogen bonding were Cys29, Asn45, Asn71, His87, Gln89, Tyr115, Asn129, Pro135, Pro136, and Ala137. *A0A0J8RAY9* exhibits both hydrogen and ionic bonding. Hydrogen bonding was seen in residues Cys43, Pro44, Gly45, Ile46, Leu47, Ser53, Phe58, Gln59, Syr271, and ionic bonding was seen in Arg215. The *E9DEI6* has hydrogen bonding interactions with the residues His5, Leu24, Ala79, Thr82, Thr127, Phe157, and Asp131. The residues in *E9CWQ6* that show hydrogen bonding are: Asn58, Asp66, Asn69, Gln72, Glu81, Leu105, and Thr237. Finally, residues Cys100, Ser128, and Leu158 were engaged in hydrogen bonding for *A0A0J8S610*. These interactions play a leading role in the complex’s overall stability and binding affinity. Furthermore, they provide the specific residues needed for maintaining the bonds between ligands and proteins (Fig. 5).

## 4. Discussion

This study identified 6 potential therapeutic targets in *C posadasii*, comprising 3 vaccine candidates and 3 drug targets. The biological significance of these targets varies considerably in their known functions and therapeutic potential. Among the drug targets, the 2 glutathione S-transferases (E9CRD4 and A0A0J8RAY9) represent particularly promising candidates given their established roles in fungal pathogenesis. Glutathione S-transferases are critical for cellular detoxification and oxidative stress resistance in fungi, and their disruption has been shown to impair virulence in other pathogenic species including *Candida albicans* and *Aspergillus fumigatus*. The catechol dioxygenase (E9CWQ6) represents a novel target class, potentially involved in aromatic compound metabolism, though its specific role in *Coccidioides* pathogenesis remains poorly characterized.

Genomic data for multiple strains of *C posadasii* were retrieved from the NCBI database, with proteomes generated for strains lacking available data. Orthology analysis using OrthoFinder facilitated the identification of core proteomes and nonhomologous proteins across strains, essential for understanding strain-specific variations and evolutionary dynamics. Subcellular localization prediction and essentiality analysis were conducted to discern the roles of proteins in pathogenesis. Cello, a machine learning-based tool, predicted the subcellular localization of proteins, highlighting their distribution across various cellular compartments. Essential genes were identified using the Geptop 2.0 web server, integrating diverse features to enhance the accuracy of prediction. Vaccine and drug targets were identified based on stringent criteria, considering protein localization, essentiality, and potential immunogenicity or drug-ability. Extracellular proteins were prioritized as vaccine targets, while metabolic pathways unique to the pathogen were explored for drug targeting. Molecular docking analysis validated the potential interactions between selected proteins and ligands, providing insights into therapeutic interventions.

The study yielded valuable insights into the genomic and proteomic landscape of *C posadasii*, with proteomic data retrieved for multiple strains. Subcellular localization prediction revealed a diverse array of proteins distributed across cellular compartments, with a subset identified as potential vaccine or drug targets. Essentiality analysis highlighted genes crucial for pathogenicity, laying the groundwork for targeted therapeutic interventions. Identification of vaccine and drug candidates highlighted promising avenues for combating coccidioidomycosis. Extracellular proteins emerged as potential vaccine targets, while metabolic pathways unique to the pathogen offered opportunities for drug targeting. Molecular docking analysis validated the potential interactions between selected proteins and ligands, providing a basis for further experimental validation.

The vaccine candidates present a more limited functional profile. While the cyanovirin-N domain-containing protein (A0A0J8S610) represents its candidacy due to its predicted secreted localization, cyanovirin-N domains are typically associated with carbohydrate binding in marine organisms,^[56]^ making their relevance to *Coccidioides* virulence lower. It might be a weaker candidate unlike other targets for the vaccine development needing significant experimental validation of biological role. E9D8Y3 represents a promising vaccine candidate as lyases involved in cell wall modification^[57]^ or metabolic processes essential for spherule development could serve as targets for neutralizing antibodies that disrupt critical fungal life cycle transitions.^[58]^ On the other hand, E9DEI6 (adhesion molecule) is one of the best vaccine target because adhesion molecules are proven successful vaccine targets.^[59]^ It’s also the best out of all candidate because calcium-dependent adhesion molecules are typically involved in host–pathogen interactions and tissue colonization, making them ideal targets for antibodies that could block fungal attachment and invasion.

The comprehensive investigation into the genomic and proteomic landscape of *C posadasii* represents a significant advancement in our understanding of coccidioidomycosis pathogenesis. By leveraging bioinformatics tools and methodologies, the study sheds light on key virulence factors and essential genes essential for the pathogen’s survival and proliferation. The identification of vaccine and drug candidates holds promise for the development of targeted therapeutics and preventive measures against valley fever.

The diverse array of proteins identified in this study underscores the complexity of *C posadasii* pathogenesis. Subcellular localization prediction revealed proteins localized to various cellular compartments, suggesting their involvement in diverse biological processes. Essentiality analysis further elucidated the roles of these proteins in pathogenicity, highlighting potential vulnerabilities for therapeutic targeting. The identification of vaccine targets, particularly extracellular proteins, presents an opportunity for developing novel immunotherapies against coccidioidomycosis. By targeting antigens exposed on the pathogen’s surface, vaccines can elicit a robust immune response, conferring protection against infection. Moreover, the exploration of metabolic pathways unique to the pathogen offers insights into potential drug targets, paving the way for the development of novel antifungal agents. Molecular docking analysis provides valuable insights into the potential interactions between selected proteins and ligands, guiding the rational design of therapeutic interventions. The validation of these interactions lays the groundwork for further experimental validation, including in vitro and in vivo studies to assess efficacy and safety.

## 5. Conclusion

In conclusion, this research represents a significant advancement in our understanding of *C posadasii* pathogenesis and the identification of potential therapeutic solutions for coccidioidomycosis. By employing a multi-faceted approach integrating genomics, proteomics, and computational biology, we have unveiled crucial insights into the virulence mechanisms of this fungal pathogen. The identification of extracellular proteins as vaccine candidates and key metabolic pathways as drug targets holds promise for the development of novel interventions against valley fever. Molecular docking simulations provide valuable guidance for the design of targeted therapeutics, facilitating the rational selection of drug candidates with favorable binding affinities. However, further experimental validation, including preclinical studies and clinical trials, will be essential to assess the efficacy and safety of these potential interventions in real-world settings. The translation of these findings into clinical practice has the potential to significantly impact the management and prevention of coccidioidomycosis, alleviating the burden of this debilitating disease on affected individuals and communities worldwide.

## Acknowledgments

Authors would like to express their appreciation to Ongoing Research Funding program (ORF-2026-885) at King Saud University, Riyadh, Saudi Arabia, for supporting this research.

## Author contributions

**Conceptualization:** Abdullah R. Alanzi.

**Data curation:** Hattan A. Alharbi, Mohammad K. Parvez, Abdelaaty A. Shahat.

**Formal analysis:** Ramzi A. Mothana, Abdelaaty A. Shahat.

**Investigation:** Abdullah R. Alanzi.

**Methodology:** Hattan A. Alharbi, Mohammad K. Parvez.

**Supervision:** Abdullah R. Alanzi.

**Validation:** Ramzi A. Mothana, Mohammad K. Parvez.

**Visualization:** Ramzi A. Mothana, Abdelaaty A. Shahat.

**Writing – original draft:** Hattan A. Alharbi, Ramzi A. Mothana.

**Writing – review & editing:** Abdullah R. Alanzi, Mohammad K. Parvez, Abdelaaty A. Shahat.










