# Supplementary material for: In silico identification and validation of vaccine and drug targets in Coccidioides posadasii through integrated genomic, proteomic, and molecular modeling approaches
Source: Medicine (Baltimore). 2026 Jun 5;105(23):e49169. doi: 10.1097/MD.0000000000049169 (PMC13246128; doi:10.1097/MD.0000000000049169)
Supplement: Supplementary file 1 [file medi-105-e49169-s001.docx]

**Table S3:** Proteins which were selected for Vaccine and Drug targets, information given in the Table S3 is retrieved from Uniprot.

| Vaccine Targets | | | | | | | | |
| --- | --- | --- | --- | --- | --- | --- | --- | --- |
| UniProt Entry | **Protein name** | | **Mass (kDa)** | | **Length** | **TMHMM (PredHel)** | | **Vaxijen** |
| E9D8Y3 | Uncharacterized Protein | | 15120 Da | | 137 | 0 | | 0.8352 |
| E9DEI6 | Uncharacterized Protein | | 22018 Da | | 197 | 0 | | 0.9051 |
| A0A0J8S610 | Cyanovirin-N domain-containing protein | | 17809 Da | | 171 | 0 | | 0.9855 |
| Drug Targets | | | | | | | | |
| Uniprot Entry | | **Protein name** | | **Mass (kDa)** | | | **Length** | |
| E9CRD4 | | Glutathione transferase | | 28595 Da | | | 252 | |
| A0A0J8RAY9 | | Glutathione S-transferase | | 31191 Da | | | 276 | |
| E9CWQ6 | | Catechol dioxygenase | | 32080 Da | | | 287 | |
