# Supplementary material for: In silico identification and validation of vaccine and drug targets in Coccidioides posadasii through integrated genomic, proteomic, and molecular modeling approaches
Source: Medicine (Baltimore). 2026 Jun 5;105(23):e49169. doi: 10.1097/MD.0000000000049169 (PMC13246128; doi:10.1097/MD.0000000000049169)
Supplement: Supplementary file 2 [file medi-105-e49169-s002.docx]

**Table S1:** Information of 5 strains of *Coccidioides posadasii* used in this research study.

| Assembly | Strain | Size | GC% | No of Proteins | |
| --- | --- | --- | --- | --- | --- |
| GCA_000151335.1 | C735 delta SOWgp | 27mb | 46.5 | | 7,229 |
| GCA_018416015.2 | Silveira | 28.2mb | 46.5 | | 8,299 |
| GCA_020976795.1 | 3796 | 28.6mb | 46.5 | | *NA* |
| GCA_020976775.1 | 2566 | 27.8mb | 46.5 | | *NA* |
| GCA_000150055.1 | RMSCC 3488 | 28.1mb | 46.5 | | 9,897 |
