# Supplementary material for: In silico identification and validation of vaccine and drug targets in Coccidioides posadasii through integrated genomic, proteomic, and molecular modeling approaches
Source: Medicine (Baltimore). 2026 Jun 5;105(23):e49169. doi: 10.1097/MD.0000000000049169 (PMC13246128; doi:10.1097/MD.0000000000049169)
Supplement: Supplementary file 3 [file medi-105-e49169-s003.docx]

**Table S2:** Below table shows the number of proteins and their locations predicted by Cello Tool.

| Location | Proteins |
| --- | --- |
| Cytoplasmic | 369 |
| Plasma Membrane | 537 |
| Mitochondrial | 331 |
| Extracellular | 150 |
| Cyto-Skeletal | 3 |
| Vacuole | 6 |
| Lysosomal | 13 |
| Nuclear | 1466 |
| Perixosomal | 2 |
