# Supplementary material for: In silico identification and validation of vaccine and drug targets in Coccidioides posadasii through integrated genomic, proteomic, and molecular modeling approaches
Source: Medicine (Baltimore). 2026 Jun 5;105(23):e49169. doi: 10.1097/MD.0000000000049169 (PMC13246128; doi:10.1097/MD.0000000000049169)
Supplement: Supplementary file 5 [file medi-105-e49169-s005.docx]

**Table4:** Vaccine and Drug candidates are given in this table, with the binding affinity , Molecular weight (Da), hydrogen bond acceptors, hydrogen bond donors, and LogP value.

| Sr No | Target | Receptor | Ligand | Binding Affinity | Molecular Weight (Da) | HBA | HBD | LogP(o/w) |
| --- | --- | --- | --- | --- | --- | --- | --- | --- |
| 1 | Vaccine target | E9D8Y3 | <CID:123631> | -7.4 | 446.91 | 6 | 1 | 3.149 |
| 2 | Vaccine target | E9DEI6 | <CID:193962> | -6.5 | 435.285 | 4 | 2 | 3.806 |
| 3 | Vaccine target | A0A0J8S610 | <CID:3045233> | -6.4 | 344.41 | 5 | 4 | 2.403 |
| 4 | Drug Target | E9CRD4 | <CID:6741> | -6.6 | 374.47 | 5 | 3 | 2.148 |
| 5 | Drug Target | A0A0J8RAY9 | <CID:214348> | -6.6 | 373.36 | 6 | 4 | 3.4571 |
| 6 | Drug Target | E9CWQ6 | <CID:11450633> | -6.0 | 339.39 | 4 | 1 | 1.1 |
